# Targeting the EGFR/PCNA Signaling Suppresses Tumor Growth of Triple-Negative Breast Cancer Cells with Cell-Penetrating PCNA Peptides

**DOI:** 10.1371/journal.pone.0061362

**Published:** 2013-04-08

**Authors:** Yung-Luen Yu, Ruey-Hwang Chou, Jia-Hong Liang, Wei-Jung Chang, Kuo-Jung Su, Yen-Ju Tseng, Wei-Chien Huang, Shao-Chun Wang, Mien-Chie Hung

**Affiliations:** 1 Graduate Institute of Cancer Biology and Center for Molecular Medicine, China Medical University, Taichung, Taiwan; 2 Department of Biotechnology, Asia University, Taichung, Taiwan; 3 The Ph.D. Program for Cancer Biology and Drug Discovery, China Medical University, Taichung, Taiwan; 4 Department of Cancer and Cell Biology, University of Cincinnati College of Medicine, Cincinnati, Ohio, United States of America; 5 Department of Molecular and Cellular Oncology, The University of Texas MD Anderson Cancer Center, Houston, Texas, United States of America; The University of Kansas Medical center, United States of America

## Abstract

Tyrosine 211 (Y211) phosphorylation of proliferation cell nuclear antigen (PCNA) coincides with pronounced cancer cell proliferation and correlates with poor survival of breast cancer patients. In epidermal growth factor receptor (EGFR) tyrosine kinase inhibitor (TKI)-resistant cells, both nuclear EGFR (nEGFR) expression and PCNA Y211 phosphorylation are increased. Moreover, the resistance to EGFR TKI is a major clinical problem in treating EGFR-overexpressing triple-negative breast cancer (TNBC). Thus, effective treatment to combat resistance is urgently needed. Here, we show that treatment of cell-penetrating PCNA peptide (CPPP) inhibits growth and induces apoptosis of human TNBC cells. The Y211F CPPP specifically targets EGFR and competes directly for PCNA tyrosine Y211 phosphorylation and prevents nEGFR from binding PCNA *in vivo*; it also suppresses tumor growth by sensitizing EGFR TKI resistant cells, which have enhanced nEGFR function and abrogated classical EGFR membrane signaling. Furthermore, we identify an active motif of CPPP, RFLNFF (RF6 CPPP), which is necessary and sufficient to inhibit TKI-resistant TNBC cell growth of orthotopic implanted tumor in mice. Finally, the activity of its synthetic retro-inverted derivative, D-RF6 CPPP, on an equimolar basis, is more potent than RF6 CPPP. Our study reveals a drug candidate with translational potential for the future development of safe and effective therapeutic for EGFR TKI resistance in TNBC.

## Introduction

Triple-negative breast cancer (TNBC) is a breast cancer subtype that is negative for estrogen receptor (ER) and progesterone receptor (PR) and epidermal growth factor receptor 2 (HER2). TNBC accounts for approximately 15–20% of all breast cancer cases and seems to be closely related to basal-like breast cancer. Patients with TNBC have a relatively poor outcome and cannot be treated with endocrine therapy or targeted therapies due to lack of related receptors [1], [2], [3]. Thus, there is a substantial need for new therapies that can target TNBC and the progression of this disease.

Epidermal growth factor receptor (EGFR) is overexpressed in TNBC. In fact, expression of EGFR is one of the defining characteristics of TNBC and a predictor of poor prognosis [4]. Clinical testing of EGFR tyrosine kinase inhibitors (TKIs) in advanced breast cancer patients demonstrated that EGFR TKIs are ineffective in treating this disease even though EGFR is overexpressed [5], [6]. EGFR also functions as a transcription factor and a tyrosine kinase that enhances cell proliferation in the nucleus [7], [8], [9], [10], [11]. For example, nuclear EGFR (nEGFR) phosphorylates and stabilizes proliferating cell nuclear antigen (PCNA) which enhances the proliferative potential of TNBC cells [7]. Furthermore, nEGFR is also a prognostic factor in human disease [12], [13], [14]. For instance, subcellular distribution of EGFR to the nucleus has been implicated in acquired resistance to cetuximab therapy in non-small cell lung cancer [15] and gefitinib (Iressa) therapy in TNBC and epithelial carcinoma [16], [17], [18]. Accumulating evidence of the functional impact of nEGFR demonstrates a need to understand the extent to which this protein contributes to cancer growth and progression as well as to the therapeutic response to EGFR-targeted therapies.

PCNA assembles in a homotrimeric ring encircling the DNA double helix and functions as a mobile sliding clamp to recruit other proteins involved in cell cycle regulation and DNA synthesis and repair [19], [20], [21]. We previously reported that nEGFR phosphorylates the chromatin-bound PCNA at tyrosine 211 (Y211), which is required to stabilize PCNA and its associated functions such as DNA synthesis and repair [7]. In addition, increased PCNA Y211 phosphorylation coincides with pronounced cell proliferation, and PCNA Y211 phosphorylation correlates better with poor survival of breast cancer patients in tumors than the total PCNA level [7]. Recently, Zhao et al. reported that phosphorylation of Y211 is a frequent event observed in human prostate cancer, and downregulation of Y211 phosphorylation by Y211F peptide in prostate cancer cells inhibits cell growth and tumor development in a xenograft mouse model [22]. These results provide proof of concept for the idea of targeting PCNA Y211 phosphorylation as an approach for prostate cancer treatment. Therefore, targeting p-Y211 PCNA could also be an effective treatment strategy for breast cancer.

To date, a growing list of transducible proteins, including modified TAT transduction domains such as TAT-p53, TAT-Smac, TAT-Src, TAT-Indip, TAT-Grb7, and TAT-PTD4-MUC1 peptides, among others, in a wide range of sizes and functional classes have been successfully used to study intracellular mechanisms and delivery *in vivo* [23], [24], [25], [26], [27], [28]. In this study, we synthesized a TAT-based Y211F cell-penetrating PCNA peptide (CPPP) that blocks Y211 phosphorylation and inhibits growth of triple-negative and EGFR TKI-resistant breast cancer cells. A shortened RF6 CPPP with the active motif of CPPP decreased tumor growth in xenograft mouse model. Our results provide evidence to support RF6 CPPP as a novel approach to target triple-negative and EGFR TKI-resistant breast cancer.

## Materials and Methods

### Ethics Statement

The study protocol was approved by the Institutional Animal Care and Use Committee of China Medical University and Hospital (No. 99-37-N).

### Cell culture, peptides, and antibodies

Human triple-negative breast cancer (TNBC) cell lines (MDA-MB 468, and MDA-MB 231) were purchased from American Type Culture Collection. All cells were maintained in DMEM/F12 (1∶1) with 10% FBS. EGFR TKI-resistant TNBC cell lines: Iressa-resistant MDA-MB 468 (IR) and Tarceva-resistant MDA-MB 468 (TR) were established by treatment with gradually increasing concentrations of these drugs for over one month. These TKI-resistant clones were cultured in the presence of 1 µM TKIs. The following peptides were synthesized by MDBio, Inc: Y211F CPPP (Y211F; YGRKKRRQRRRGTFALRFLNFFTK); AK10 CPPP (AK10; YGRKKRRQRRRGALRFLNFFTK); TF10 CPPP (TF10; YGRKKRRQRRRGTFALRFLNFF); TN8 CPPP (TN8; YGRKKRRQRRRGTFALRFLN); RK8 CPPP (RK8; YGRKKRRQRRRGRFLNFFTK); RF6 CPPP (RF6; YGRKKRRQRRRGRFLNFF); D-RF6 CPPP (D-RF6; YGRKKRRQRRRGRFLNFF); Scrambled peptides for Y211F (Scramble; YGRKKRRQRRRGFLYTNKLFRTAF); Scrambled peptides for RF6 (Scramble; YGRKKRRQRRRGLNFFRF). The following antibodies were purchased from commercial companies: anti-α-tubulin (Sigma); anti-lamin B (Calbiochem); anti-EGFR (Lab Vision); anti-PCNA (Santa Cruz); anti-ERK1/2 (Cell Signaling); anti-pERK1/2 (Cell Signaling); anti-AKT (Cell Signaling); anti-pAKT (Cell Signaling); anti-Histone H4 (Santa Cruz); anti-caspase-3 (Santa Cruz). The anti-phospho-Y211 PCNA antibody was raised against a phosphorylated synthetic peptide and purified with the phosphopeptide column by LTKBiol, Inc.

### Cell extraction, immunoprecipitation and Western blotting

Cell extraction, immunoprecipitation and Western blotting were performed as described previously [7], [29].

### Cell viability assay

Cell viability was determined by WST-1 (4-[3-(4-Iodophenyl)-2-(4-nitrophenyl) -2H-5-tetrazolio]-1,3-benzene disulfonate) assay (Roche). Cells were incubated with or without TKI or CPPP. After culturing for another 24 h, one-tenth volume of WST-1 was added at 4 h before harvest, and the absorbance was detected at 450 nm. Cell viability was normalized by the absorbance from the cells without treatment.

### Apoptosis assay

For apoptosis assay using annexin V staining, cells treated with the CPPP for 24 h and the cells were harvested and resuspended in the binding buffer and then incubated with FITC-conjugated Annexin V and propidium iodide (PI), according to the manufacturer's protocol (BD Biosciences). The percentage of apoptotic cells were assessed by flow cytometry.

### Confocal microscopy

Confocal microscopy was performed as previously described [29].

### Animal Model

For subcutaneous implantation, MDA-MB 468 cells (1×10^7^ in 100 µL of sterile Dulbecco's phosphate-buffered saline; PBS) were inoculated into nude mice by subcutaneous injection into the flanks. Each group contained 4 mice that were inoculated with tumor cells on both flanks (n = 8). When the tumors were palpable, mice were grouped randomly into 3–4 groups with 4 mice in each group. The mice were then treated with control vehicle, control scrambled peptide, Y211F CPPP, RF6 CPPP, or D-RF6 CPPP, by intratumoral injection. For orthotopic implantation, tumor xenografts were established by injection of Iressa-resistant MDA-MB 468 (IR) cells (1×10^7^ resuspended in a 1∶1 mixture of PBS and Matrigel (BD Biosciences) in a total volume of 100 µL) into the mammary fat pad on either side of each 6–8-week old female severe combined immunodeficient (SCID) mice, respectively. When the tumors were palpable, the mice bearing IR tumor xenografts were treated with control vehicle, control scrambled peptide, D-RF6 CPPP (200 nmol/mouse) by intraperitoneal injection. Tumor growth was monitored 3 times/week for five weeks by measuring tumor perpendicular diameters. Tumor volume (V) was calculated using the following formula: V = length×diameter^2^×0.5.

### Statistical analysis

Data from each assay were presented as means ± SD from at least three independent experiments (*n* = 3). Statistical analysis between 2 groups were determined by the Student's *t* test. *p*<0.05 was considered significantly different.

## Results

### Inhibition of Y211 phosphorylation of PCNA suppresses cell growth and induces apoptosis in TNBC cells

**Figure 1:**
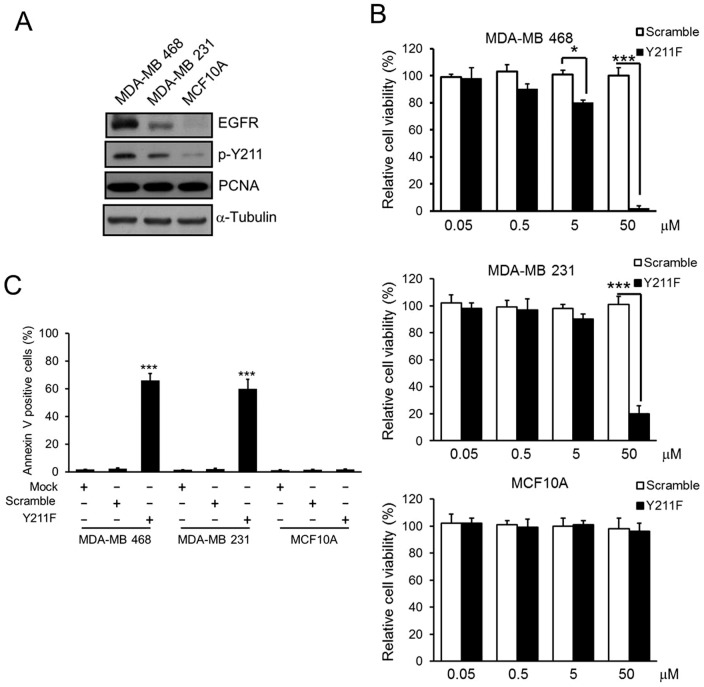
The phosphorylation of PCNA at Y211 and the cytotoxic effects of CPPP in normal breast cell line and TNBC cells. (A) Total cell lysate from MCF10A normal breast cell line or MDA-MB 468 and MDA-MB 231 TNBC cell lines were extracted, the protein levels of EGFR and total PCNA were determined by immunoblotting (IB). The Y211 phosphorylation of PCNA was examined by immunoprecipitation (IP) with anti-p-Y211 antibody and followed by IB against PCNA. (B) Each TNBC cell line was treated with indicated concentration of scrambled (Scramble) or Y211 CPPP (Y211F) peptides for 24 h. The relative cell viability was measured by WST-1 assay. (C) The percentage of the apoptotic cells from each TNBC cell line after 24 h treatment with 15 µM of Mock (PBS), Scramble and Y211F were determined by flow cytometry of Annexin V-positive cells. The bars indicate mean ± S.D. *, p<0.05; ***, p<0.001 by *t*-test.

**Figure 2:**
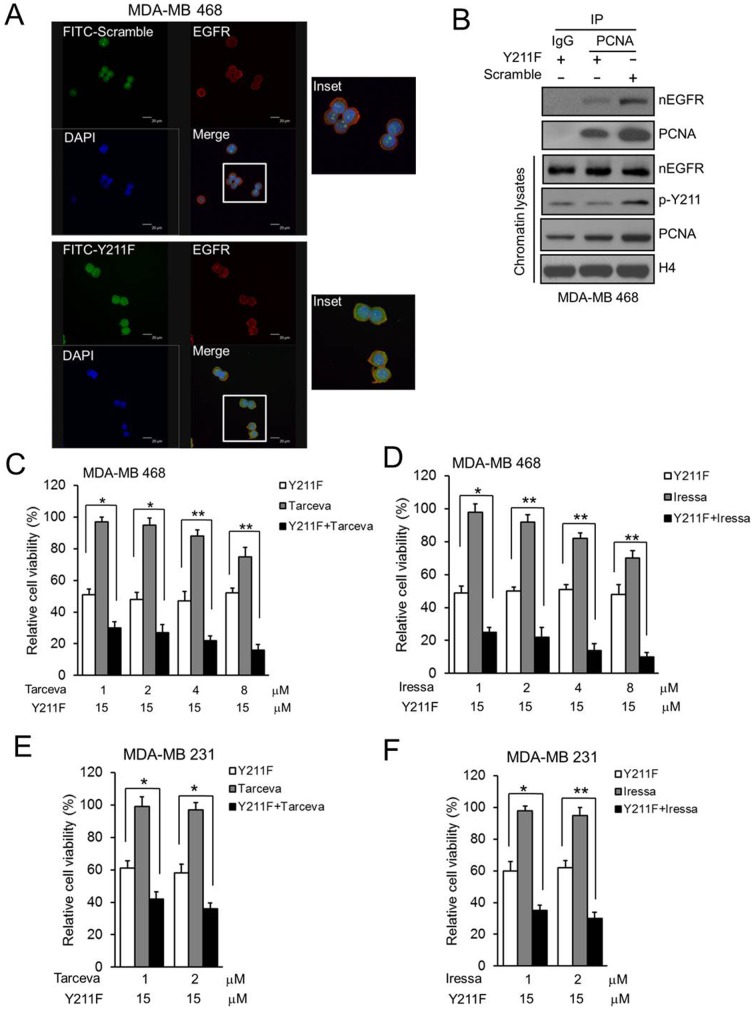
The effect of CPPP on PCNA Y211 phosphorylation and EGFR TKIs. (A) MDA-MB 468 cells were treated with 30 µM FITC-labeled scrambled (FITC-Scrambled; top) or Y211F CPPP (FITC-Y211F; bottom) for 12 h. Subsequently, cells were fixed, permeabilized, and stained with anti-EGFR antibody or DAPI. The fluorescent images were observed under a confocal microscopy. The FITC-labeled peptide (green), EGFR (red), and nucleus (blue) are shown. (B) MDA-MB 468 cells were treated with 30 µM scrambled (Scramble) or Y211F CPPP (Y211F) for 24 h. The chromatin lysates were extracted and IP with IgG or anti-PCNA antibody and separated by SDS-PAGE followed by IB for nEGFR, Y211-phosphorylated PCNA (p-Y211) and PNCA in MDA-MB 468 cells. (C–F) Both MDA-MB 468 or MDA-MB 231 cells were treated with 15 µM Y211F CPPP or 1∼8 µM EGFR TKI (Tarceva or Iressa) alone, or Y211F CPPP and TKI combined for 24 h. The relative cell viability after each treatment was then determined.

Nuclear translocation of EGFR stabilizes PCNA and controls its functions including DNA synthesis and repair through phosphorylation at Y211 [7]. We found that both EGFR and PCNA Y211 phosphorylation from total cell lysates were much higher in MDA-MB 468 and MDA-MB 231 TNBC cells compared to MCF10A mammary epithelial cells derived from normal breast tissue (Fig. 1A). To disrupt PCNA stability on the chromatin, MDA-MB 468 and MDA-MB 231 TNBC cells were treated with the synthetic TAT-based Y211F CPPP that suppresses PCNA Y211 phosphorylation. This peptide consists of 12 amino acids flanking the region of the Y211 with the tyrosine (Y) residue replaced by a phenylalanine (F). The Y211F CPPP suppressed cell viability in both MDA-MB 468 and MDA-MB 231 cells in a dose-dependent manner but had no effect on MCF10A, even at the 50 µM concentration that killed more than 80% of cancer cells (Fig. 1B). In addition, apoptotic response measured by Annexin V-positive cells after the treatment of the Y211F CPPP was observed only in MDA-MB 468 and MDA-MB 231 cells but not in MCF10A (Fig. 1C). Both the TAT-based scrambled peptide and Y211F CPPP penetrated the cell membrane for entry into the cytosol and nucleus compartments of cells but only the Y211F CPPP specifically targeted EGFR (Fig. 2A) and prevented nEGFR from binding PCNA to compete for PCNA tyrosine Y211 phosphorylation and stability on the chromatin in MDA-MB 468 cells (Fig. 2B).

### Y211F CPPP enhances the effect of EGFR TKI and sensitizing EGFR TKI-resistant TNBC cells

**Figure 3:**
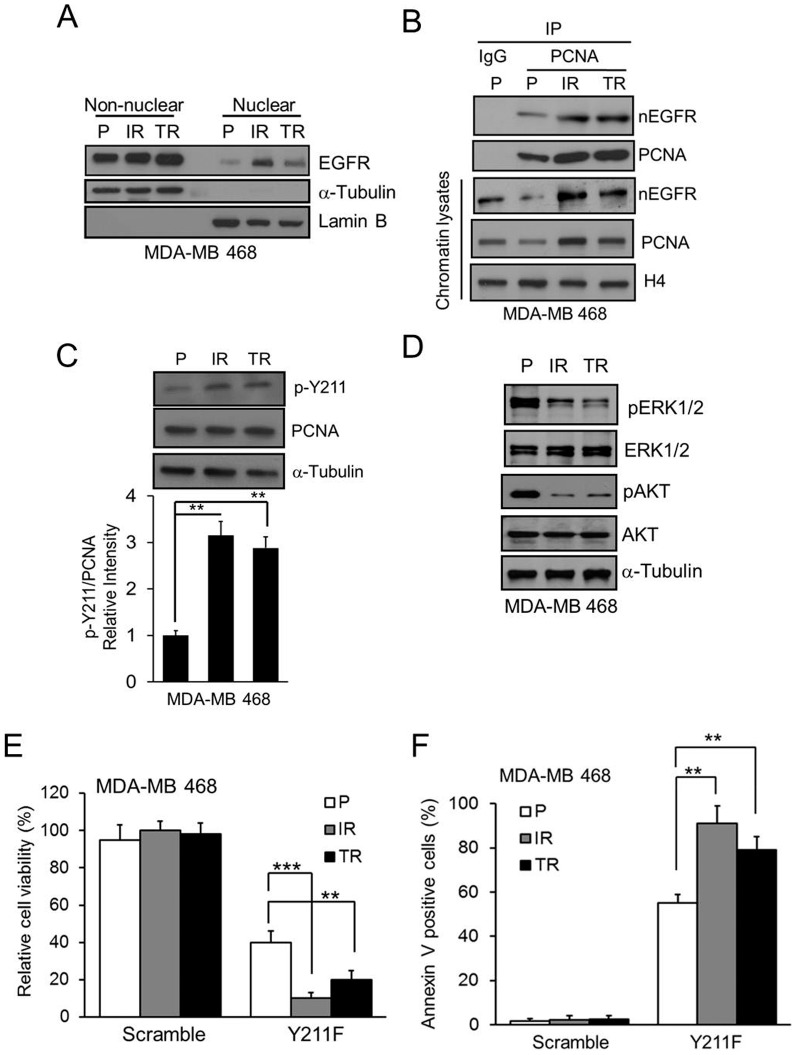
The cytotoxic effect of CPPP in TKI-resistant TNBC cells. (A) Cell lysate from non-nuclear and nuclear fractions were isolated from parental (P), Iressa-resistant (IR) or Tarceva-resistant (TR) MDA-MB 468 cells. The protein level of EGFR in each fraction was examined by IB. α-tubulin and lamin B were used as the markers of non-nuclear and nuclear fraction, respectively. (B) The association between nEGFR and PCNA in chromatin lysate from P-, IR- and TR-TNBC cells were verified by IP with IgG or anti-PCNA antibody. (C) The amount of p-Y211 on PCNA in each TNBC line was examined. A comparison of the relative intensity p-Y211 in IR- or TR-TNBC cells to the parental TNBCs is shown at the bottom. (D) Total cell lysate from P-, IR- and TR-TNBC cells were isolated. The protein level of indicated proteins were examined by IB. (E–F) The P-, IR-, or TR-TNBC cells were treated with 15 µM Y211F CPPP for 24 h and the relative cell viability (E) and Annexin V-positive cells (F) were determined. The bars indicate mean ± S.D. **, p<0.01; ***, p<0.001 by *t*-test.

To determine if CPPP increases the sensitivity of TNBC cells to EGFR TKI, we further treated the EGFR-positive MDA-MB 468 and MDA-MB 231 TNBC cells with a combination of the Y211F CPPP and clinically used EGFR TKIs, Tarceva (Erlotinib) and Iressa (Gefitinib). We found that the Y211F CPPP enhanced the effect of both TKIs at more physiological doses in these TNBC cells (Fig. 2C–2F). As drug resistance of cancer cells is one of the important reasons for therapeutic failure or cancer recurrence, we asked whether the Y211F CPPP is also effective in the EGFR TKI-resistant TNBC cells. To this end, we established two more EGFR TKI-resistant TNBC cell lines: Iressa-resistant MDA-MB 468 (IR) and Tarceva-resistant MDA-MB 468 (TR). The results showed that the amounts of nEGFR (Fig. 3A), binding to PCNA (Fig. 3B), stability of PCNA (Fig. 3B) and Y211 phosphorylated PCNA (Fig. 3C) were enhanced in both the IR and TR cells compared with the parental (P) cells. Importantly, IR and TR cells were attenuated the classical EGFR membrane signaling including downstream ERK1/2 and AKT pathways (Fig. 3D) and were more sensitive to Y211F CPPP treatment than the parental cells (Fig. 3E and 3F), suggesting that the Y211F CPPP has the potential to treat the IR or TR TNBC patients.

### The RFLNFF motif of CPPP is biologically active in TNBC cells

**Figure 4:**
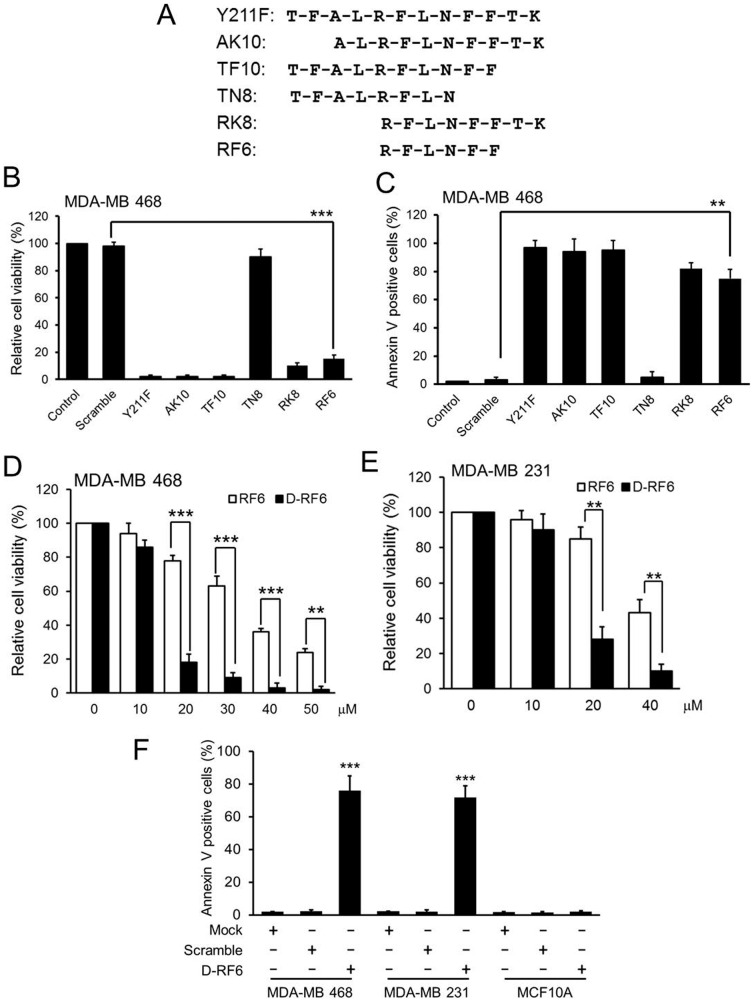
The cytotoxic effect of different CPPP derivatives in TNBC cells. (A) The schematic illustrates the amino acid sequence of each CPPP derivative. (B–C) MDA-MB 468 cells were individually treated with 50 µM of each CPPP derivative for 24 h, and then the relative cell viability (B) and Annexin V-positive cells (C) were determined. (E–F) MDA-MB 468 (E) or MDA-MB 231 (F) cells were treated with indicated concentrations of RF6 CPPP or D-RF6 CPPP for 24 h, and the relative cell viability was examined. (G) The percentage of the apoptotic cells from each TNBC cell line after 24 h treatment with 15 µM of Mock (PBS), Scramble and D-RF6 were determined by flow cytometry of Annexin V-positive cells. The bars indicate mean ± S.D.; **, p<0.01; ***, p<0.001 by *t*-test.

To map the critical and minimal effective region of the CPPP, we synthesized a series of CPPP derivatives with different lengths (Fig. 4A) and determined how each of the peptide derivatives affects the viability of TNBC cells. Except for TN8 CPPP (TN8; TFALRFLN), all tested CPPP derivatives were effective in suppressing cell viability (Fig. 4B) and induce apoptosis (Fig. 4C). The minimal effective peptide was RF6 CPPP (RF6; RFLNFF), which indicates that the active motif RFLNFF within the 12 amino residues of CPPP is critical (Fig. 4B and 4C). In addition, since most mammalian proteases cannot cleave a D form retro-inverted peptide, this type of modification of biologically active motifs has been used to increase the stability of peptide-based drug candidates [30]. We also converted the RF6 CPPP to a D form RF6 (D-RF6) CPPP and found this conversion enhanced the growth suppression and apoptosis activity of the unmodified peptide in both MDA-MB 468 and MDA-MB 231 TNBC cells (Fig. 4E–4G). These results indicate that we can use the reduced and modified D form retro-inverted peptide as a therapeutic candidate for TNBC treatment meriting further development.

### Tumor targeting of CPPP *in vivo*

**Figure 5:**
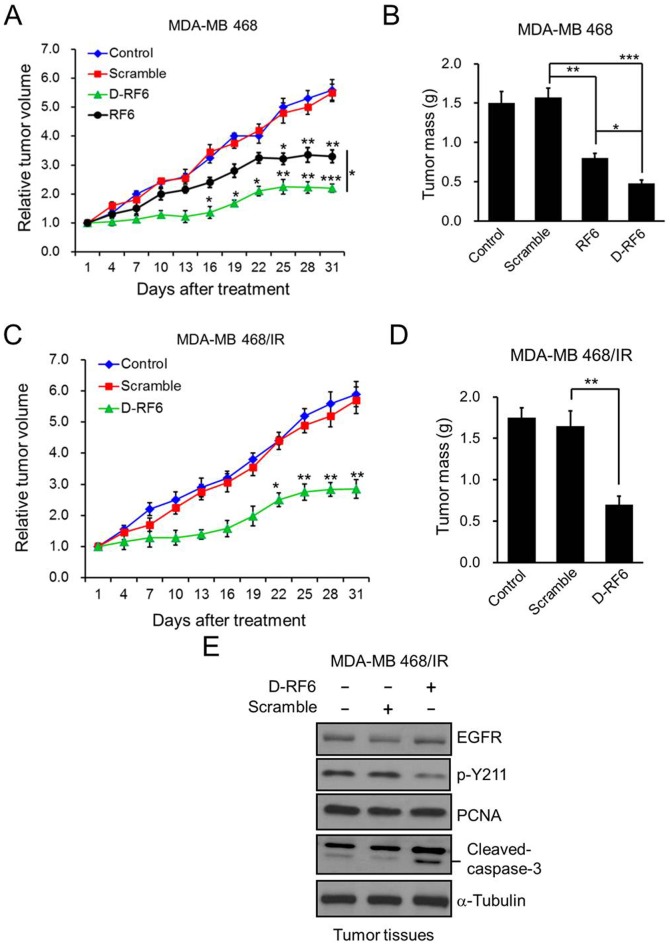
The effect of CPPP on tumor growth *in vivo*. (A) Approximately 1×10^7^ MDA-MB 468 cells were subcutaneously injected into the flanks of nude mice. When the tumors were palpable, mice were randomly divided into 4 groups and treated with PBS (Control), scrambled peptide for RF6 (Scramble), RF6 CPPP, or D-RF6 CPPP (200 nmol/mouse) by intratumoral injection. Tumor volume was measured the tumor volume at the indicated time point. (B) The weight of each of the harvested tumors from (A) was measured after treatment. (C) Approximately 1×10^7^ Iressa-resistant MDA-MB 468 (MDA-MB 468/IR) cells were injected into the mammary fat pad on either side of SCID mice. When the tumors were palpable, mice were randomly divided into 3 groups and treated with PBS (Control), scrambled peptide for D-RF6 (Scramble), or D-RF6 CPPP (200 nmol/mouse) by intratumoral injection. Tumor volume was measured the tumor volume at the indicated time point. (D) The weight of each of the harvested tumors from (C) was measured after treatment. The curves and bars indicate mean ± S.D.; *, p<0.05; **, p<0.01; ***, p<0.001 by *t*-test. (E) Extracted tumor tissue lysates from (D), the protein levels of EGFR, p-Y211, PCNA and cleaved-caspase-3 were determined by IB.

**Figure 6:**
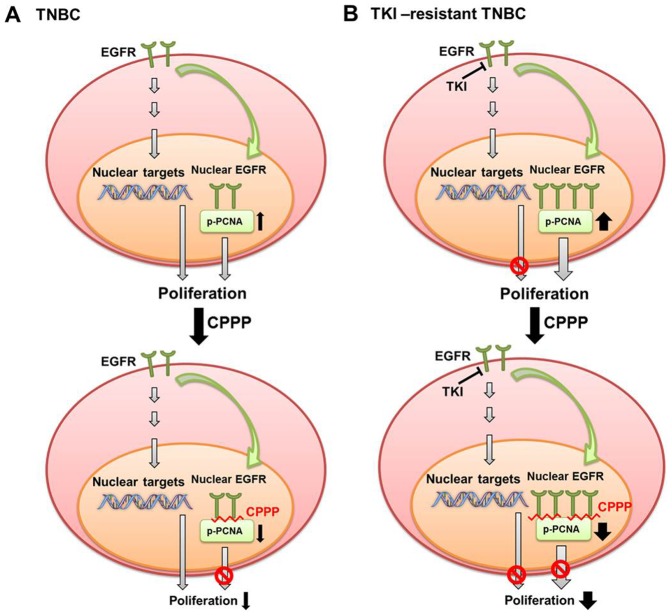
A proposed model of CPPP in TNBC treatment. The schematic illustrates the potential mechanism of CPPP in treatment of TKI-sensitive and -resistant TNBC. (A) In TNBC, both the classical EGFR membrane signaling and nuclear EGFR-mediated phosphorylation of PCNA at Y211 exist for cell proliferation. CPPP disrupts the nuclear EGFR-mediated PCNA phosphorylation at Y211, leading to suppression of proliferation. (B) In EGFR TKI-resistant TNBC, EGFR TKIs abrogate the classical EGFR membrane signaling, and thus elevate nuclear translocation of EGFR to phosphorylate more PCNA at Y211, triggering more second compartment of proliferation. CPPP attenuates more nuclear EGFR-mediated cell proliferation through repression of Y211 phosphorylation in the presence of EGFR TKIs.

To further validate the anti-tumor effect of CPPP *in vivo*, we established a xenograft animal model by subcutaneous injection of MDA-MB 468 cells into the flanks of nude mice. When the tumors were palpable, mice were randomized into 3 groups and treated with PBS (Control), scrambled peptide (Scramble), or CPPP (Y211F, RF6 or D-RF6) by intratumoral injection. Tumor volume was measured at each time interval. As shown in Fig. 5 and S1, mice treated with Y211F CPPP (Fig. S1A), RF6 CPPP (Fig. 5A) and D-RF6 CPPP (Fig. 5A) had significantly reduced tumor volume compared with those treated with control or scrambled peptides. At the end of the experiment, all mice were sacrificed, and their tumors were isolated and weighed. The CPPP-treated (Y211F CPPP, RF6 CPPP and D-RF6 CPPP) mice had significantly smaller tumor mass than those in the control or scrambled peptide group (Fig. S1B and 5B). The activity of D-RF6 CPPP, on an equimolar basis, appeared to be more potent than Y211F CPPP in suppressing the tumor growth of TNBC and reducing tumor volume in mice. Further, in addition to the subcutaneous implantation, we utilize syngeneic orthotopic implantation model to investigate the role of D-RF6 CPPP in suppresses breast tumor growth especially the therapeutic effect against EGFR TKI-resistant TNBC. Indeed, as shown in Fig. 5C and 5D, mice treated with D-RF6 CPPP had significantly reduced tumor volume and mass compared with those treated with control or scrambled peptides, suggesting the potential application of the D-RF6 CPPP for TKI-resistant TNBC therapy. Moreover, to further demonstrate that D-RF6 CPPP targets PCNA *in vivo*, an extracted tumor tissue from Fig. 5D and shown that PCNA Y211 phosphorylation is inhibited and also inducing apoptosis signaling e.g. cleaved caspase-3 increased *in vivo* (Fig. 5E). Based on our studies, we proposed a model demonstrating the how CPPP can be used for treating both TNBC and the acquired EGFR TKI-resistant type. In TNBC, cell proliferation depends on the classical EGFR membrane signaling and in part on nuclear EGFR-mediated proliferation via phosphorylation of PCNA at Y211. Treating TNBC with CPPP, e.g., Y211F CPPP, disrupts nuclear EGFR-mediated PCNA phosphorylation at Y211 and destabilizes PCNA, leading to suppression of proliferation (Fig. 6A). In EGFR TKI-resistant TNBC, the classical EGFR membrane proliferating signaling is attenuated in the presence of EGFR TKIs, and elevated nEGFR (a characteristic of TKI-resistant cells) phosphorylates more PCNA at Y211, triggering more nEGFR proliferating signaling (Fig. 6B). CPPP is likely to repress cell proliferation more effectively in EGFR TKI-resistant than non-resistant TNBC as a result of higher PCNA Y211 phosphorylation and loss of classical EGFR membrane signaling in the presence of EGFR TKIs in the resistant cells.

## Discussion

PCNA is a known cell proliferation marker during the S and G2 phases of the cell cycle in breast cancer cells [31], [32]. In addition, higher PCNA expression has been shown to be a poor prognosis marker for cancer patients, particularly in breast cancer [31], [33], [34], [35] http://www.pnas.org/cgi/content/full/103/51/19472?maxtoshow=&HITS=10&hits=10&RESULTFORMAT=1&andorexacttitle=and&andorexacttitleabs=and&andorexactfulltext=and&searchid=1&FIRSTINDEX=0&sortspec=relevance&volume=103&firstpage=19472&resourcetype=HWCIT - B5#B5. In breast cancer patients, a higher expression level of PCNA correlated with poor survival rate [34]. However, there are contradictory reports questioning the fidelity of PCNA as a clinical marker in tumor progression [36], [37], [38], [39], [40], [41]. Indeed, PCNA has been viewed as a non-specific proliferation marker because of its role in DNA repair [42]. In addition, increased PCNA Y211 phosphorylation coincides with pronounced cell proliferation, and Y211 phosphorylation in tumors correlated better with poor survival of breast cancer patients than the total PCNA level. The discrepancies among the previous reports may be due to its phosphorylation status (p-Y211).

In our current study, we show that Y211 phosphorylation of PCNA is frequently higher in TNBC cells (Fig. 1A). In each of the TNBC cell lines tested, inhibition of the phosphorylation by Y211F CPPP inhibited cell viability (Fig. 1B). In addition to the anti-proliferation effect, downregulation of PCNA Y211 phosphorylation also resulted in cell death (Fig. 1C). Thus, the induction of cell death may be linked to the pathway mediating the response to cell cycle arrest-induced apoptosis. Alternatively, PCNA Y211 phosphorylation may also function in regulating cell viability during cell proliferation. Discriminating between these possibilities will require further understanding the underlying molecular mechanisms and the signaling pathway in PCNA-mediated cell death.

Clinical testing of EGFR TKIs in breast cancer patients demonstrated that EGFR TKIs are not effective in treating TNBC even though EGFR is overexpressed [5], [6]. Interestingly, we found that CPPP sensitizes TNBC cells to TKI-mediated cell growth inhibition and can overcome EGFR TKI resistance (Fig. 3). We further showed that the most active motif of CPPP, RFLNFF, is sufficient to inhibit cell growth *in vitro* (Fig. 4) and attenuate tumor growth in mice (Fig. 5). In addition, since peptide-based drugs are often susceptible to degradation by proteases *in vivo*, the biological activity of a cell-penetrating peptide depends directly on its stability in blood serum. Therefore, the D-RF6 CPPP, which is a synthetic D form retro-inverted derivative, has translational potential for the future development of safe and effective therapeutics against EGFR TKI-resistant TNBC.

## Supporting Information

Figure S1 **The effect of Y211F CPPP on tumor growth in vivo.** (A) Approximately 1×10^7^ MDA-MB 468 cells were subcutaneously injected into the flanks of nude mice. When the tumors were palpable, mice were randomly divided into 3 groups and treated with PBS (Control), scrambled peptide for Y211 (Scramble), or Y211F CPPP (200 nmol/mouse) by intratumoral injection. Tumor volume was measured the tumor volume at the indicated time point. (B) The weight of each of the harvested tumors was measured after treatment. The curves and bars indicate mean ± S.D.; *, p<0.05; **, p<0.01; ***, p<0.001 by *t*-test.
(TIF)
