# Supplementary figures and images for: Targeting the EGFR/PCNA Signaling Suppresses Tumor Growth of Triple-Negative Breast Cancer Cells with Cell-Penetrating PCNA Peptides
Source: PLoS One. 2013 Apr 8;8(4):e61362. doi: 10.1371/journal.pone.0061362 (PMC3620387; doi:10.1371/journal.pone.0061362)

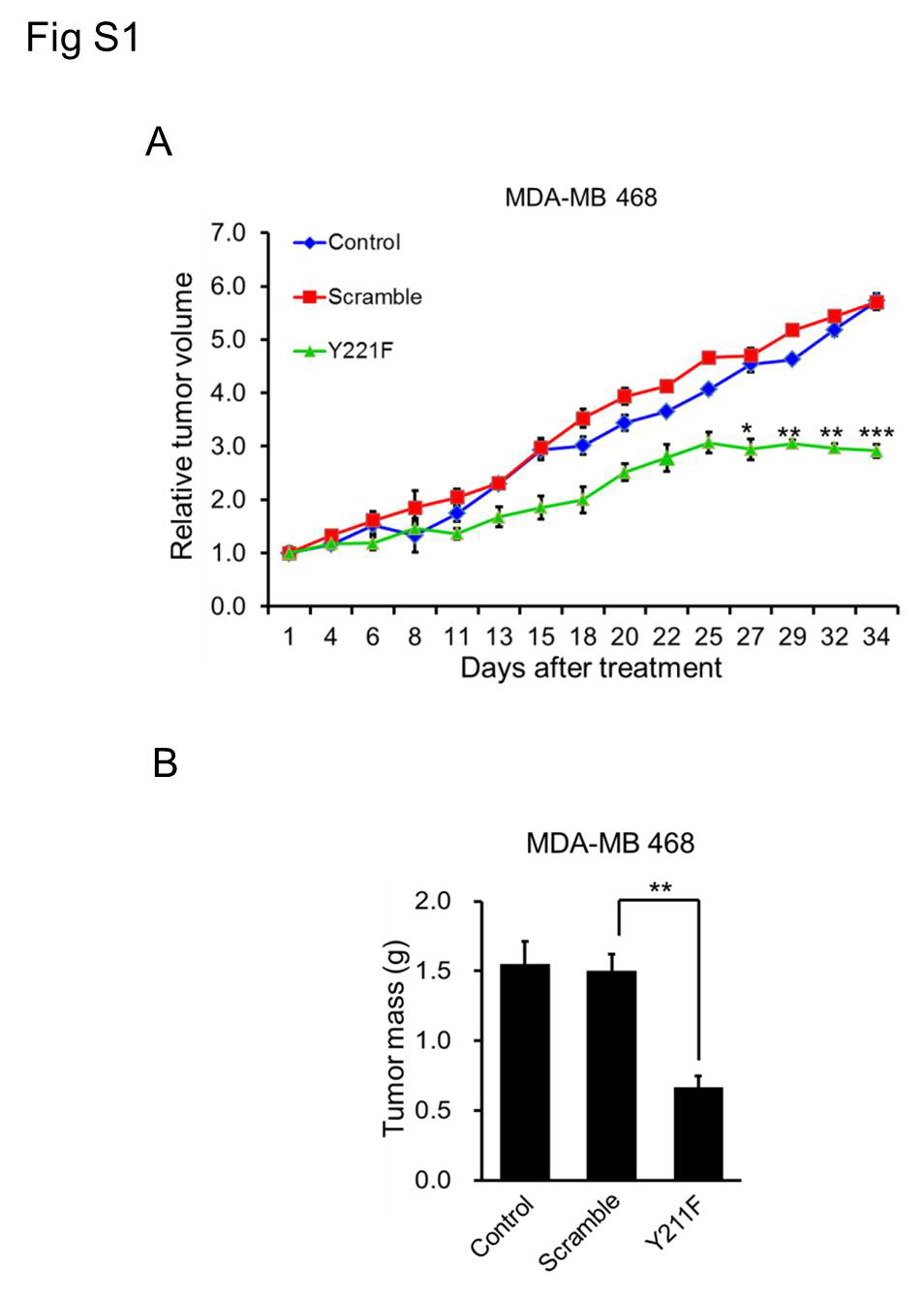

Supplement: Figure S1 — The effect of Y211F CPPP on tumor growth in vivo. (A) Approximately 1×10^7^ MDA-MB 468 cells were subcutaneously injected into the flanks of nude mice. When the tumors were palpable, mice were randomly divided into 3 groups and treated with PBS (Control), scrambled peptide for Y211 (Scramble), or Y211F CPPP (200 nmol/mouse) by intratumoral injection. Tumor volume was measured the tumor volume at the indicated time point. (B) The weight of each of the harvested tumors was measured after treatment. The curves and bars indicate mean ± S.D.; *, p<0.05; **, p<0.01; ***, p<0.001 by t-test. (TIF) [file pone.0061362.s001.tif]
